# Membrane lipid rafts are required for AMPA receptor tyrosine phosphorylation

**DOI:** 10.3389/fnsyn.2022.921772

**Published:** 2022-10-31

**Authors:** Takashi Hayashi

**Affiliations:** ^1^Biomedical Research Institute, National Institute of Advanced Industrial Science and Technology, Tsukuba, Ibaraki, Japan; ^2^Department of Molecular Neurobiology and Pharmacology, Graduate School of Medicine, The University of Tokyo, Tokyo, Japan

**Keywords:** AMPA receptor, Src family protein tyrosine kinase, palmitoylation, tyrosine phosphorylation, membrane, lipid rafts

## Abstract

Membrane lipid rafts are sphingolipids and cholesterol-enriched membrane microdomains, which form a center for the interaction or assembly of palmitoylated signaling molecules, including Src family non-receptor type protein tyrosine kinases. Lipid rafts abundantly exist in neurons and function in the maintenance of synapses. Excitatory synaptic strength is largely controlled by the surface expression of α-amino-3-hydroxy-5-methyl-4-isoxazole propionate (AMPA) receptors in the mammalian brain. AMPA receptor endocytosis from the synaptic surface is regulated by phosphorylation of the GluA2 subunit at tyrosine 876 by Src family kinases. Here, I revealed that tyrosine phosphorylated GluA2 is concentrated in the lipid rafts fraction. Furthermore, stimulation-induced upregulation of GluA2 tyrosine phosphorylation is disrupted by the treatment of neurons with a cholesterol-depleting compound, filipin III. These results indicate the importance of lipid rafts as enzymatic reactive sites for AMPA receptor tyrosine phosphorylation and subsequent AMPA receptor internalization from the synaptic surface.

## Introduction

Glutamate is the major excitatory neurotransmitter in the mammalian central nervous system (CNS). Glutamate receptors (GluRs) majorly mediate fast excitatory synaptic transmission in the brain (Seeburg, 1993; Hollmann and Heinemann, 1994; Mori and Mishina, 2003). Excitatory synaptic strength is largely controlled by dynamic changes of α-amino-3-hydroxy-5-methyl-4-isoxazole propionate (AMPA)-type GluRs (AMPA receptors) expression on the synaptic surface, especially by a balance between rapid insertion of intracellular AMPA receptors into the surface and internalization of surface-expressing AMPA receptors in a stimulation-dependent manner (Collingridge et al., 2004; Kessels and Malinow, 2009; Huganir and Nicoll, 2013). AMPA receptors are composed of four subunits, GluA1–4 (also known as GluR1–4, GluRA–D, or GluRα1–4). Major functional AMPA receptors in the adult CNS comprise GluA1/GluA2- and GluA2/GluA3-containing receptors (Shepherd and Huganir, 2007; Heine et al., 2008; Henley and Wilkinson, 2016; Diering and Huganir, 2018). The GluA2 subunit is the primary determinant during endocytosis from synapses and post-endocytic endosomal sorting (Anggono and Huganir, 2012; Diering and Huganir, 2018).

Previous studies have revealed that trafficking processes of AMPA receptors are regulated by several post-translational protein modifications of the AMPA receptor subunits, including reversible phosphorylation, and *S*-palmitoylation of them (Jiang et al., 2006; Anggono and Huganir, 2012; Lu and Roche, 2012; Parkinson and Hanley, 2018). Among those dynamic modifications, tyrosine phosphorylation of the GluA2 subunit enhances AMPA receptors internalization from the synaptic surface (Hayashi and Huganir, 2004; Gladding et al., 2009; Hu et al., 2010; Yong et al., 2020). We have shown that Src family protein tyrosine kinases (PTKs) catalyze tyrosine phosphorylation of GluA2 at tyrosine 876 (Hayashi and Huganir, 2004). GluA2 tyrosine phosphorylation induces an exchange of GluA2-binding scaffolding proteins from glutamate receptor-interacting proteins 1/2 (GRIP1/2) to protein interacting with C-kinase (PICK1), which sequentially drives endocytosis of surface AMPA receptors (Hayashi and Huganir, 2004; Yong et al., 2020). Furthermore, the interaction of GluA2 with the synaptic protein BRAG2, a guanine-nucleotide exchange factor (GEF) for the coat-recruitment GTPase Arf6, occurs in a GluA2 tyrosine phosphorylation-dependent manner, which induces the AMPA receptor endocytosis in long-term synaptic depression (Scholz et al., 2010).

Src family PTKs are palmitoylated and myristoylated at their N-terminal regions (Neet and Hunter, 1996). Covalent attachments of saturated fatty acids, 16-carbon containing palmitate and 14-carbon containing myristate, on the non-receptor type PTKs generally increase their affinity to membrane fraction and induce their translocation from cytosolic region to membrane (Nadolski and Linder, 2007; Resh, 2016). Furthermore, accumulating evidence indicates that these protein lipidations enable signaling molecules to limitedly localize in a specific lipid microdomain in the cellular membrane, termed lipid rafts (Simons and Vaz, 2004; Turner et al., 2005; Pike, 2006). Lipid rafts are primarily sphingolipids and cholesterol-enriched dynamic membrane microdomains, which have been proposed as a center for the interaction or assembly of palmitoylated signaling molecules, including Src family PTKs, G-proteins, and various membrane receptors (Kasahara et al., 2000; Moffett et al., 2000; Foster et al., 2003; Fujita et al., 2007). The brain is the organ with the highest enrichment in lipids, including sphingolipids and cholesterol (Aureli et al., 2015; Schnaar, 2016). Lipid rafts abundantly exist in the dendrites of neurons and function in the maintenance of synapses as well as in the stabilization of surface AMPA receptors (Hering et al., 2003). Here, I present that the lipid rafts structure is essential for GluR agonist-induced tyrosine phosphorylation of GluA2, which exhibits the importance of lipid rafts as enzymatic reactive sites for Src family PTKs-mediated AMPA receptor modification.

## Materials and methods

### Drugs and antibodies

Filipin III and Optiprep™, a non-ionic iodixanol-based density gradient medium, were purchased from Sigma-Aldrich (St. Louis, MO, USA). Anti-GluA2 N-terminal monoclonal antibody (MAB397, Chemicon, Temecula, CA, USA), anti-GluA2 phospho tyrosine 876 polyclonal antibodies (JH3410, Hayashi and Huganir, 2004; Hu et al., 2010; Yong et al., 2020), anti-Fyn monoclonal antibody (F19720, Transduction Laboratories, Lexington, KY, USA), and anti-Caveolin1 polyclonal antibodies (N-20, sc-894, Santa Cruz Biotechnology, Santa Cruz, CA, USA) were used for the experiments.

### Primary neuron culture and stimulation

High-density cultured cortical neurons were prepared on poly-L-lysine coated culture dishes of 60 mm diameter dish as described previously (Hayashi and Huganir, 2004). Cultured cortical neurons (10^6^ cells per lane) were used between 3 and 4 weeks [at 24, 29, 31, and 34 days *in vitro* (DIV)] after plating for biochemical experiments. For stimulation of AMPA receptors, cortical culture neurons at 25–26 days DIV were washed three times with 3 ml of extracellular artificial cerebrospinal fluid (aCSF) solution containing 15 mM HEPES, pH 7.5, 140 mM NaCl, 5 mM KCl, 1 mM MgCl_2_, 5 mM glucose, 0.5 mM EDTA, and 0.12% NaHCO_3_. The extracellular solution was replaced by a 2 ml aCSF solution containing 5 μM TTX to inhibit neuronal activity. Then, cultured cortical neurons were treated with or without 10 μg/ml Filipin III and incubated in the same buffer at 37°C for 10 min before stimulation with 100 μM AMPA for 10 min. An amount of 2.5 mg/ml of Filipin III dissolved in DMSO was prepared at the time of use as a stock solution, and 8 μl Filipin III (final 10 μg/ml) solution or DMSO was added to extracellular solutions of cultured cortical neurons (Gimpl et al., 1997; Foster et al., 2003; Lambert et al., 2006).

### Analysis of proteins

Neurons were lysed in 1.2 ml buffer containing 50 mM Tris–HCl, pH8.0, 1% Triton X-100, 150 mM NaCl, 20 mM EDTA, 1 mM Na_3_VO_4_, 100 μg/ml phenylmethylsulfonyl fluoride (PMSF), 2 μg/ml aprotinin, 1 μg/ml pepstatin, and 0.5 μg/ml leupeptin. Homogenates were centrifuged at 15,000 × *g* for 20 min at 4°C. Lysates were separated by 7.5% SDS-PAGE, followed by Western blotting with each antibody. Blocking and washing solutions for immunoblotting were 5% skim milk and 0.1% Tween-20 in tris-buffered saline (TBS) (50 mM Tris–HCl, pH 7.5, and 150 mM NaCl) and TBS containing 0.05% Triton X-100, respectively. Reactive bands were visualized with the electrochemiluminescence (ECL). Chemiluminescent images were acquired using the ImageQuant LAS 4000 mini imager (GE Healthcare, Tokyo, Japan). The phosphorylation ratio was estimated by comparing the intensity of each band: tyrosine phosphorylated GluA2 to total GluA2 protein amount in the cultured neurons and control without filipin III treatment was defined as 100%.

### Density gradient fractionation

**FIGURE 1:**
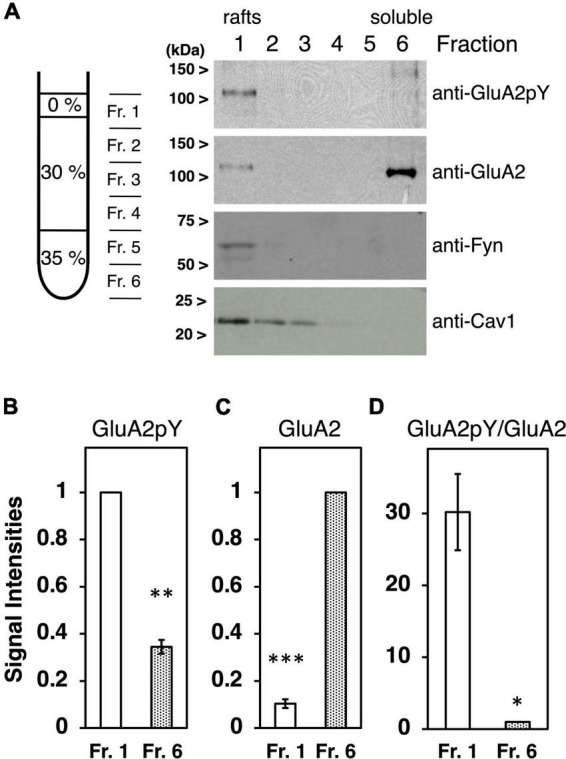
Characterization of tyrosine phosphorylated GluA2 in lipid rafts fraction. **(A)** Proteins expressed in cultured cortical neurons were separated by Optiprep-density gradients from the least dense fraction 1 (Fr. 1) to the densest Fr. 6. Representative immunoblots of tyrosine phosphorylated GluA2 (anti-GluA2pY) and GluA2, Fyn, or Caveolin-1 (Cav1) proteins expression are shown. **(B)** Comparison of tyrosine phosphorylated GluA2 in Fr. 1 (white bar) and Fr. 6 (dotted bar). Band intensities are normalized to Fr. 1 (*n* = 3). **(C)** Comparison of GluA2 protein amount in Fr. 1 (white bar) and Fr. 6 (dotted bar). Band intensities are normalized to Fr. 6 (*n* = 3). **(D)** Ratio of tyrosine phosphorylated GluA2 to GluA2 protein amount in Fr. 1 (white bar) and Fr. 6 (dotted bar). Values are normalized to Fr. 6 (*n* = 3). Error bars represent SEM. *p < 0.05, ^**^p < 0.01, ^***^p < 0.001, *t*-test.

Cultured neurons at 27–35 DIV were washed twice with 1.0 ml cold phosphate-buffered saline (PBS) and scraped in 1.5 ml buffer containing 20 mM Tris–HCl, pH7.5, 250 mM Sucrose, 50 mM NaCl, 1 mM DTT, 1 mM Na_3_VO_4_, a cocktail of protease inhibitors. Collected neurons were mechanically homogenized on ice by five passages through a 22G syringe needle, followed by centrifugation at 1,000 × *g* for 10 min at 4°C. The pellets were resuspended in 0.5 ml buffer and repeatedly homogenized by five passages through the 22G syringe needle and centrifugation. Combined 2.0 ml supernatants were adjusted to 35% Optiprep in a total of 4.8 ml and overlaid with 2.5 ml of each 30, 20, and 5% Optiprep prepared in the same buffer. The gradients were spun at 40,000 rpm for 3 h at 4°C in an SW41 rotor using a Beckman ultracentrifuge. The interface 5 and 20% boundary (900 μl) was collected, followed by the addition of two-thirds volume (600 μl) of the same buffer containing 1.25% Triton X-100 (final 0.5%), and lysed for 60 min at 4°C with rotation. Then, lysates were adjusted to 35% Optiprep in a totally of 3.6 ml and overlaid with 7.5 ml of 30% Optiprep and 1.5 ml of 0% Optiprep prepared in the same buffer containing 0.1% Triton X-100, 1 mM Na_3_VO_4_. The gradients were spun at 40,000 rpm for 5 h at 4°C in the SW41 rotor. Six fractions were collected from top to bottom of centrifuge tubes (Smart et al., 1995; Lafont et al., 1998, 1999; Oliferenko et al., 1999; Lindwasser and Resh, 2001; Inoue et al., 2004; Nakagawa et al., 2005; Pike et al., 2005). An amount of 2 ml of each fraction was diluted by the addition of equal volume (2 ml) PBS and concentrated by ultrafiltration with Centricon30 (Amicon). Fractionated proteins were analyzed by Western blotting with each antibody. For the estimation of signal intensities, the rafts fraction (Fr. 1) in Figure 1B and the soluble fractions (Fr. 6) in Figures 1C,D were defined as 100%.

### Statistical analysis

Statistical analyses were performed using Prism 9 (GraphPad Software) and Excel (Microsoft). Student’s *t*-tests or ANOVA followed by Tukey *post-hoc* test was used to compare groups. All data are expressed as mean ± standard error of the mean (SEM).

## Results

### Tyrosine phosphorylated GluA2 is concentrated in the lipid rafts fraction

To clarify the submembrane domain where tyrosine phosphorylated AMPA receptors localize, subcellular membrane fractionation was performed using Optiprep-density gradients. The method for the preparation of lipid rafts involves the extraction of crude membrane fraction from cultured cortical neurons in 0.5% Triton X-100, followed by separation of the low-density raft membranes in an Optiprep-density gradient (Figure 1A). Localization of Fyn PTK, as representative of palmitoylated Src family PTKs, and lipids raft-associated protein Caveolin-1 (Nabi and Le, 2003; Michel and Bakovic, 2007) were examined to confirm accurate isolation of lipid rafts domain (Figure 1A). Tyrosine phosphorylated GluA2 signals were majorly detected in fraction 1, corresponding to the lipid rafts fraction (Figure 1B, *n* = 3, *p* = 0.0020). Whereas most GluA2 proteins were detected in the detergent soluble fraction indicated as fraction 6 (Figure 1C, *n* = 3, *p* = 0.0004), tyrosine-phosphorylated GluA2 was remarkably concentrated in the lipid rafts fraction (Figure 1D, *n* = 3, *p* = 0.0315). GluA2 and its tyrosine phosphorylation signals retain at the undetectable level in Fractions 2–5.

### Reduced GluA2 tyrosine phosphorylation by disruption of cholesterol-enriched membrane fraction

**FIGURE 2:**
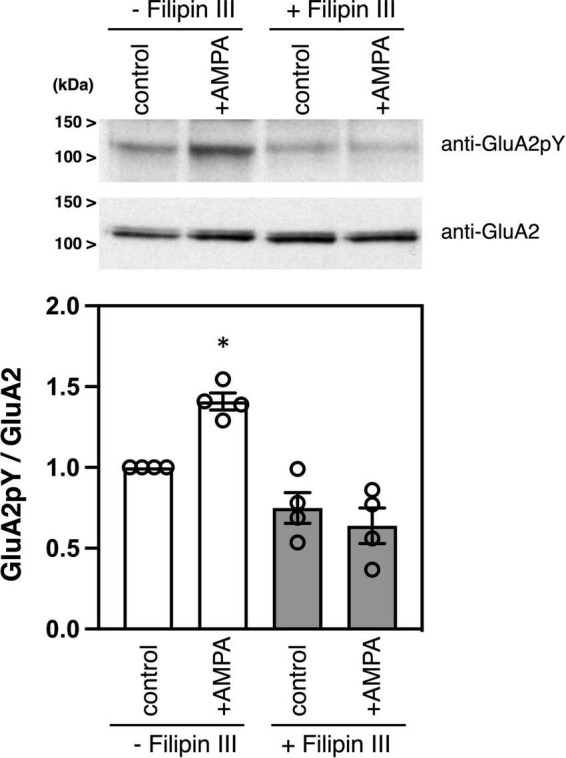
Effects of filipin III treatment on AMPA-induced tyrosine phosphorylation of GluA2 in cultured cortical neurons. Cultured cortical neurons were treated with DMSO or 10 μg/ml Filipin III for 10 min, followed by stimulation with 100 μM AMPA for 10 min. Cell lysates from cultured cortical neurons were immunoblotted with anti-GluA2CpY or anti-GluA2 antibodies to quantify tyrosine phosphorylated GluA2 and GluA2 protein expression. Typical blots as representatives are shown **(Top)**. The ratio of tyrosine phosphorylated GluA2 to GluA2 protein amounts is statistically analyzed **(Bottom)**. Error bars represent SEM. *p < 0.05, compared with control.

Filipin is an antibiotic polyene, which forms a complex with cholesterol in membranes and is widely used to disrupt lipid rafts in cells (Gimpl et al., 1997; Foster et al., 2003). Filipin is a mixture of four components, filipin I, II, III, and IV, and filipin III is the major component of the filipin complex, which provides a useful tool for studying the role of lipid rafts in cellular mechanisms (Lambert et al., 2006). Treatment of cultured neurons with filipin III blocks cholesterol-mediated endocytosis of membrane proteins (Auriac et al., 2010). As we previously showed (Hayashi and Huganir, 2004), stimulation of cultured cortical neurons with an agonist AMPA increases phosphorylation of GluA2 C-terminus at Tyr876 without affecting GluA2 protein expression amounts (Figure 2). Pretreatment of cortical neurons with filipin III significantly suppressed the AMPA-induced GluA2 tyrosine phosphorylation in cortical neurons [Figure 2, *n* = 4 each, ANOVA, *F*_(3, 15)_ = 19.79, *p* = 0.0042].

## Discussion

Src family PTKs-mediated phosphorylation of the AMPA receptor and subsequent exchange of GluA2-binding proteins play crucial roles in the endocytosis of GluA2-containing synaptic AMPA receptors (Hayashi and Huganir, 2004; Gladding et al., 2009; Hu et al., 2010; Yong et al., 2020). Src family PTKs are associated with gangliosides-containing lipid rafts prepared from the rodent brain (Kasahara et al., 1997, 2000). Our previous study showed that stimulation with AMPA induces the activation of AMPA receptor-associated Src family PTKs in cultured neurons (Hayashi et al., 1999). The experimental data presented here revealed that tyrosine phosphorylated-AMPA receptors are concentrated in lipid rafts (Figure 1). Moreover, GluR agonist stimulation-induced tyrosine phosphorylation of AMPA receptors occurs in a lipid rafts-dependent manner (Figure 2). Treatment with filipin III should disrupt the accumulation of AMPA receptors and Src family PTKs in the lipid rafts, which prevent subsequent Src family PTKs-mediated tyrosine phosphorylation of GluA2. Collectively, these results suggest that lipid rafts may provide a place for effective AMPA receptor tyrosine phosphorylation and they consequently enable receptor internalization from the synaptic surface.

We have previously shown that Src family PTKs-mediated tyrosine phosphorylation of GluA2 is detected in the cerebral cortex, hippocampus, and cerebellum of the intact mouse brain to a greater or lesser degree (Hayashi and Huganir, 2004). Moreover, stimulation with glutamate, AMPA, or *N*-methyl-D-aspartate (NMDA) upregulates GluA2 tyrosine phosphorylation and induces the following AMPA receptor internalization in cultured cortical neurons. The existence of signaling pathways is predictable, including AMPA receptors-, NMDA receptors-, and mGluRs-mediated activation of downstream Src family PTKs, whereas crosstalk among these signaling still remains unclear. Here, I showed that the lipid rafts are required for the most essential AMPA receptor-mediated tyrosine phosphorylation of GluA2 as a place of phosphorylation reaction. Future analysis will reveal the detailed upstream signaling, which regulates the activation and localization of Src family PTKs in the lipid rafts.

Covalent attachments of the most abundant saturated fatty acid, palmitate, to integral membrane receptors as well as to signaling molecules such as Src family PTKs are supposed to increase proteins’ affinity to lipid rafts (Linder and Deschenes, 2007; Fukata et al., 2016). Many neuronal receptors and ion channels are regulated by their reversible modification of the palmitoylation and depalmitoylation cycle (Kang et al., 2008; Shipston, 2011; Borroni et al., 2016; Hayashi, 2021a). In various modifications evolutionarily established in the vertebrate glutamatergic excitatory synapses, the most probable mechanism that forces AMPA receptors to translocate into lipid rafts microdomain is the palmitoylation of the receptors themselves (Hayashi et al., 2005; Matt et al., 2019; Hayashi, 2021a,b).

**FIGURE 3:**
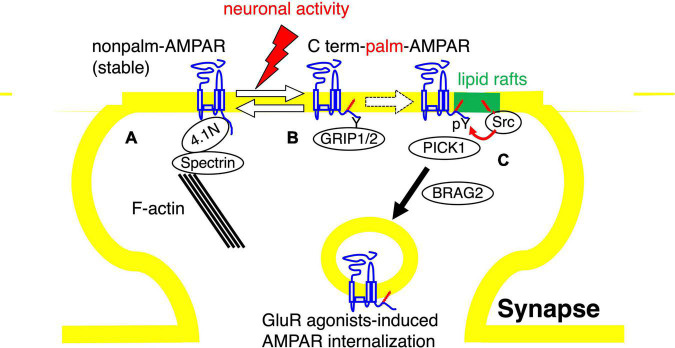
Model of the role of AMPA receptor palmitoylation in lipid rafts. Diagram of the role of AMPA receptor palmitoylation and tyrosine phosphorylation. **(A,B)** The balance between palmitoylated and de-palmitoylated forms of the AMPA receptor can be regulated by glutamate receptor agonists treatment, suggesting that synaptic activity can regulate the steady-state level of AMPA receptor palmitoylation. De-palmitoylation of the AMPA receptor on the C-terminal site increases the receptors’ affinity for 4.1N, which stabilizes AMPA receptors at the synaptic surface by the interaction of 4.1N with F-actin and spectrin **(A)**. In contrast, the palmitoylated forms of surface AMPA receptors are less stably associated with the synaptic membrane and are more susceptible to neuronal activity-induced internalization **(B)**. **(C)** The palmitoylated AMPA receptors are assumed to translocate to the synaptic membrane lipid rafts, where the palmitoylated Src family protein tyrosine kinases are concentrated. Phosphorylation of GluA2 at Tyr876 by Src family protein tyrosine kinases induces the exchange of AMPA receptor-binding proteins from GRIP1/2 to PICK1 and the receptor interaction with BRAG2, which drive endocytosis of surface AMPA receptors.

These findings offer a possible model that links tyrosine phosphorylation and palmitoylation of AMPA receptors as follows. Depalmitoylated AMPA receptors are trapped on synaptic action fibers through their binding with the 4.1N-spectrin protein complex and these unmodified AMPA receptors show stable surface expression (Figure 3A). Neuronal activity-induced palmitoylation of AMPA receptors should serve as a trigger for the translocation of the receptors to lipid rafts domain (Figure 3B). There, lipid rafts-enriched palmitoylated Src family PTKs phosphorylate GluA2 at Tyr876, followed by an exchange of GluA2-binding scaffolding proteins from GRIP1/2 to PICK1 and interaction with BRAG2, which accelerate AMPA receptor endocytosis (Figure 3C). Appropriate membrane localization of receptor proteins generally ensure dynamic regulation of the receptor functions in neurons. Our studies suggest the significance of crosstalk between two distinct signaling pathways, tyrosine phosphorylation and palmitoylation of AMPA receptors in compartmentalized synapses.

## Data availability statement

The original contributions presented in the study are included in the article. Further inquiries can be directed to the corresponding author.

## Ethics statement

The animal study was reviewed and approved by the Institutional Review Committees of Graduate School of Medicine, The University of Tokyo and National Institute of Advanced Industrial Science and Technology (AIST).

## Author contributions

The author confirms being the sole contributor of this work and has approved it for publication.
